# Method for isolation and molecular characterization of extracellular microvesicles released from brain endothelial cells

**DOI:** 10.1186/2045-8118-10-4

**Published:** 2013-01-10

**Authors:** Arsalan S Haqqani, Christie E Delaney, Tammy-Lynn Tremblay, Caroline Sodja, Jagdeep K Sandhu, Danica B Stanimirovic

**Affiliations:** 1 National Research Council of Canada, Human Health Therapeutics Portfolio, 100 Sussex Drive, Ottawa, ON, K1A 0R6, Canada

**Keywords:** Exosomes, Proteomics, Blood–brain barrier, Drug delivery, Mass spectrometry, Microvesicles, Endothelial cells, CNS, Biomarkers, Receptor-mediated transcytosis

## Abstract

**Background:**

In addition to possessing intracellular vesicles, eukaryotic cells also produce extracellular microvesicles, ranging from 50 to 1000 nm in diameter that are released or shed into the microenvironment under physiological and pathological conditions. These membranous extracellular organelles include both exosomes (originating from internal vesicles of endosomes) and ectosomes (originating from direct budding/shedding of plasma membranes). Extracellular microvesicles contain cell-specific collections of proteins, glycoproteins, lipids, nucleic acids and other molecules. These vesicles play important roles in intercellular communication by acting as carrier for essential cell-specific information to target cells. Endothelial cells in the brain form the blood–brain barrier, a specialized interface between the blood and the brain that tightly controls traffic of nutrients and macromolecules between two compartments and interacts closely with other cells forming the neurovascular unit. Therefore, brain endothelial cell extracellular microvesicles could potentially play important roles in ‘externalizing’ brain-specific biomarkers into the blood stream during pathological conditions, in transcytosis of blood-borne molecules into the brain, and in cell-cell communication within the neurovascular unit.

**Methods:**

To study cell-specific molecular make-up and functions of brain endothelial cell exosomes, methods for isolation of extracellular microvesicles using mass spectrometry-compatible protocols and the characterization of their signature profiles using mass spectrometry -based proteomics were developed.

**Results:**

A total of 1179 proteins were identified in the isolated extracellular microvesicles from brain endothelial cells. The microvesicles were validated by identification of almost 60 known markers, including Alix, TSG101 and the tetraspanin proteins CD81 and CD9. The surface proteins on isolated microvesicles could potentially interact with both primary astrocytes and cortical neurons, as cell-cell communication vesicles. Finally, brain endothelial cell extracellular microvesicles were shown to contain several receptors previously shown to carry macromolecules across the blood brain barrier, including transferrin receptor, insulin receptor, LRPs, LDL and TMEM30A.

**Conclusions:**

The methods described here permit identification of the molecular signatures for brain endothelial cell-specific extracellular microvesicles under various biological conditions. In addition to being a potential source of useful biomarkers, these vesicles contain potentially novel receptors known for delivering molecules across the blood–brain barrier.

## Background

Brain endothelial cells (BEC) lining the brain capillaries are sealed by tight junctions and exhibit a specialized molecular and functional phenotype referred to as the blood–brain barrier (BBB). The BBB functions as a physical and enzymatic barrier and employs polarized transport systems to control the exchange of nutrients and macromolecules between the blood and the brain [1]. BECs are tightly integrated with other neighbouring cells, pericytes and astrocytes; astrocytes also communicate with neurons acting as a liaison for endothelial–neuronal coupling (the neurovascular unit; NVU). The luminal, blood-facing surface of BEC is endowed by a thick and dynamic glycocalyx involved in sensing the microenvironment and interactions with blood-borne cells. With the surface area of ~20 m^2^ in the human brain, BECs are a potential source of diagnostic/prognostic blood-accessible biomarkers characteristic of brain pathologies.

Whereas the BBB is a hindrance for delivery of therapeutics, especially macromolecules, to brain targets, specific BEC receptors that undergo receptor-mediated transcytosis (RMT) have recently been exploited for the development of ‘Trojan horses’ – molecular ligands to these receptors that can ‘piggy-back’ therapeutics across the BBB. The current spectrum of known BBB receptors that undergo RMT is limited, and only a few, including the transferrin receptor (TFRC) [2,3], insulin receptor (INSR) [4,5] and low density lipoprotein receptor-related protein 1 (LRP1) [6,7] have been used for brain delivery of macromolecules with varying success [7,8]. Mechanisms for the RMT process remain poorly understood; despite the surge in literature on intracellular sorting processes leading to receptor endocytosis and recycling, the nature of ‘transcytosing vesicles’ of the BBB remains obscure.

Most eukaryotic cells secrete a mixed population of extracellular microvesicles (EMVs). The EMVs are released either through exocytosis of multivesicular bodies (MVBs) forming 50-100 nm-diameter exosomes or through shedding of plasma membranes forming 100-1000 nm-diameter shedding vesicles or ectosomes [9-11]. EMVs have been isolated using differential centrifugation methods [12,13] from cultured supernatants and body fluids including cerebrospinal fluid . EMVs originating from different cells and tissues have been analysed using electron microscopy and various molecular methods, including proteomics [14], and the results of these analyses have been compiled as the database of proteins, miRNAs and lipids known as ExoCarta [15]. EMVs are released by various CNS cells [16-18] and endothelial cells [19], including fetal brain endothelium undergoing angiogenic sprouting [20].

EMVs contain RNA and proteins that are *specific* to the original cell type. For example, tumor-derived exosomes usually contain tumor –specific antigens as well as certain immunosuppressive proteins such as FasL, TRAIL, or TGF-β [9,21]. This cell-derived specificity and accessibility from body fluids [13] has made EMVs an attractive source of biomarkers for transcriptomic and proteomic studies. BBB-specific EMVs that are shed or secreted into the blood could be a source of biomarkers specific for CNS disorders.

Various studies have now demonstrated that EMVs are a general vehicle for cell-cell communication [10,11]. EMVs carry cell-specific protein and RNA cargo and horizontally transfer these molecules into the target cell, resulting in a rapid change in transcriptome and proteome of the target cell. A similar function of BBB-derived EMVs in the cross-talk among cells of the NVU could be envisaged, in view of recently-described role of EMVs as communication vehicles among the various parenchymal cells of the CNS [16,22,23].

**Figure 1:**
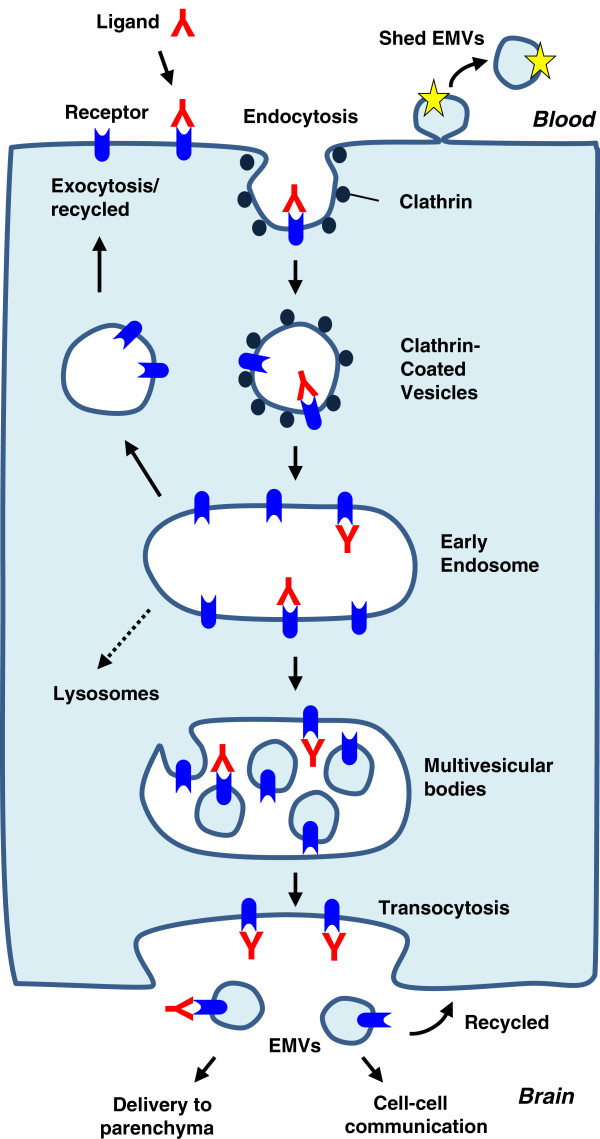
**Proposed functions of extracellular microvesicles (EMVs) at the blood–brain barrier.** EMVs ‘shed’ from the luminal membranes of BEC into the circulation contain unique molecules (as indicated by star) that potentially can be used as CNS-specific markers. Ligand binding to receptor-mediated transcytosis (RMT) receptor on the luminal surface leads to receptor-mediated endocytosis. The ligand/receptor complex is then sorted through the endocytic pathway into multivesicular bodies (MVBs) and is externalized on the abluminal side in abluminal EMVs. The EMVs can communicate with cells in the brain, including neurons and astrocytes through protein-protein surface interactions followed by transfer of RNA/protein molecules. A similar process may occur in the opposite direction, resulting in RMT receptor recycling, or ‘transfer’ of parenchymal exosomes into the systemic circulation.

We propose that EMVs derived from BECs have the potential to be (i) a source of BEC/CNS specific biomarkers; (ii) communication vesicles within neurovascular unit, and (iii) ‘transcytosing vesicles’ containing specific RMT receptors. These hypothesized functional roles for BEC EMVs are illustrated in Figure 1. This study provides initial supporting evidence for these proposed roles through analyses of molecular signatures of BEC EMVs using sensitive mass spectrometry (MS)-based proteomics protocols.

## Methods

### HBEC cultures

The immortalized human brain microvascular endothelial cells, HCMEC/D3 [24], were used in this study and are referred to as HBEC throughout the manuscript. HCMEC/D3 cell line was obtained from Dr. Pierre Olivier Couraud (Cochin Institute, Université Paris DescartesINSERM. The cells were grown in a humidified atmosphere of 5% CO_2_/95% O2 at 37°C in EBM-2 basal medium (Lonza, Walkersville, MD, USA), supplemented with one quarter of a SingleQuot kit (Lonza) and 2% fetal bovine serum in flasks coated with 100 μg/ml rat tail collagen type I (BD Canada, Mississauga, ON,Canada), diluted in 20 mM acetic acid. Cells from passages 30 to 34 were used. EMV production was done in serum-free conditions since serum has endogenous EMVs and serum molecules can non-specifically bind to HBEC-EMVs. To prepare for EMV isolation, cells were grown until confluence, washed at least three times with a buffered-saline solution and then incubated in serum-free medium for at least 1 d to obtain a sufficient amount of EMVs. While this protocol was optimized for HBEC, any mammalian cell type can be used as a starting sample for EMV isolation.

### Isolation of EMVs from HBEC

EMV isolation method was adapted from [13]. Typically 100 mL of cultured media was used by pooling from multiple dishes. The media was centrifuged at 300 ×g for 10 min at 4°C to remove any intact cells, followed by a 2,000 ×g spin for 20 min at 4°C to remove dead cells and finally a 10,000 ×g spin for 30 min at 4°C to remove cell debris. The media was then transferred to ultracentrifuge tubes and centrifuged at 100,000 ×g for at least 60 min at 4°C in Optima TLX ultracentrifuge with 60 Ti rotor (Beckman Coulter, Mississauga, Canada). The supernatant containing EMV-free media was removed and the pellets containing EMVs plus proteins from media were resuspended in PBS. The suspension was centrifuged at 100,000 ×g for at least 60 min at 4°C to collect final EMV pellets. Typically this method provided enough exosomes to be analyzed at least seven times by gel-free nanoLC-MS/MS methods (FASP, DR) or 1-3 times by gel-based methods (SDS-PAGE, Gel-LC-MS/MS or Western blotting).

### Proteomics methods

Three methods were used and compared for isolating proteins from EMVs: (i) detergent removal (DR), (ii) filtered-aided sample preparation (FASP) [25] and (iii) 1D-SDS-PAGE (gel-LC). For the DR and gel-LC methods, EMVs were dissolved in 50 mM Tris–HCl (pH 8.5), 0.2% SDS by boiling for 10 min. The samples were reduced (4 mM DTT for 10 min at 95°C) and alkylated (10 mM iodoacetamide, 30 min at room temperature in dark) and divided for DR and gel-LC analysis. For DR, SDS was removed using detergent removal spin columns (Pierce, Rockford, IL, USA) by washing against 50 mM Tris–HCl (pH 8.5) and the samples were digested overnight using trypsin (Promega, Madison, WI, USA) at 37°C for nanoLC-MS/MS analysis. For gel-LC, samples were separated on one-dimensional SDS-PAGE and stained with Coomassie blue to identify the proteins. The entire lane was cut into ten sequential bands. Each band was de-stained and was in-gel digested using trypsin at 37°C for nanoLC-MS/MS analysis.

For FASP method, EMVs were reduced in 3.5% SDS, 100 mM Tris–HCl, 100 mM DTT by boiling for 10 min. A 6.6-volume of urea solution (8 M urea, 100 mM Tris–HCl, pH 8.5) was added to the sample and they were transferred to pre-wetted Amicon-30 spin columns (Millipore, Billerica, MA, USA) and spun as per manufacturer’s instructions. The proteins were washed three times with the urea solution, alkylated (10 mM iodoacetamide, 30-60 min at room temperature in dark), and then washed four times with the urea solution and four times with 50 mM ammonium bicarbonate. The samples were digested using trypsin at 37°C and the peptides were eluted for nanoLC-MS/MS analysis.

### NanoLC-MS/MS and data analysis

The digested proteins were acidified with acetic acid (5% final concentration) and analyzed on a reversed-phase nanoAcquity UPLC (Waters, Milford, MA, USA) coupled to LTQ Orbitrap ETD mass spectrometer (ThermoFisher, Waltham, MA, USA). The analysis involved injection and loading of the desired aliquot of the sample onto a 300 μm I.D. × 0.5 mm 3 μm PepMaps® C18 trap (ThermoFisher) followed by eluting onto a 100 μm I.D. × 10 cm 1.7 μm BEH130C18 nanoLC column (Waters) using a gradient from 0% - 20%% acetonitrile (in 0.1% formic) in 1 min, 20% - 46% in 60 min, and 46% - 95% in 1 min at a flow rate of 400 nL/min. The eluting peptides were ionized into the mass spectrometer by electrospray ionization (ESI) for MS/MS using collision-induced dissociation (CID) for fragmentation of the peptide ions. Data was acquired on ions with mass/charge (m/z) values between 400 and 2,000 with 1.0 s scan duration and 0.1 s interscan interval. All MS/MS spectra were obtained on 2+, 3+, and 4+ ions. The raw data was converted to mzXML format and peak lists were submitted to a probability-based search engine, Mascot version 2.2.0 (Matrix Science Ltd., London, UK) [26]. The initial database utilized was a composite of forward and reverse Uniprot-Swiss-Prot *Homo sapiens* protein database (July 2012). Unmatched peptides were subsequently searched against the entire Uniprot-Swiss-Prot database (July 2012). Searches were performed with a specified trypsin enzymatic cleavage with one possible missed cleavage. False-positive rate (FPR) in Mascot searching was calculated as follows:

(1) $$\text{FPR}=\left(2\times \text{Nrev}\right)/\left(\text{Nrev}+\text{Nfwd}\right),$$

where Nrev is the number of peptides identified (after filtering) from the reverse-database, and Nfwd is the number of peptides identified (after filtering) from the forward database. To maximize the number of peptides and keep the FPR <0.5%, ion scores >40, parent ion tolerance of < 0.1 Da, a fragment ion tolerance of < 0.2 Da, and minimal number of missed cleavages were chosen (≤1). As an independent statistical measure of peptide identification, Peptide Prophet probabilities were also measured. All identified peptides had p ≥ 0.90. To measure the MS signal, intensities of all the ions in the MS run were extracted from the mzXML files using MatchRx software as described previously [27]. MS signal from a group of proteins was obtained by summing intensities of ions (peptides) associated with these proteins. Total MS signal was calculated by summing intensities of all the ions in the MS run.

### Detection of FC5 in EMVs using western blotting analysis or LC-MRM

For Western blotting, EMV proteins were extracted by boiling 5-10 min in Laemmli buffer (BioRad, Hercules, CA, USA) containing fresh 5%% beta-mercaptoethanol (Sigma-Aldrich, St. Louis, MO, USA). Protein extracts were resolved on a 12% discontinuous SDS-PAGE and either silver stained or electrophoretically transferred onto nitrocellulose membranes (Millipore, Nepean, Canada). Membranes were blocked in 5% non-fat dry milk powder in TBST buffer (10 mM Tris, pH 7.4, 150 mM NaCl, 0.02% Tween-20) for 2 h. Anti-V_H_H rabbit polyclonal antibody (Biogen Idec, Cambridge MA, USA) was diluted at 1:1000 in 2.5% milk in TBST and incubated with the membranes for 18 h at 4°C. Membranes were washed 4 times in TBST and then incubated for 1 h with goat anti-rabbit-HRP (Sigma-Aldrich), diluted 1:8000 in TBST. Membranes were washed 4 times with TBST and then developed by ECL Plus Chemiluminescent Substrate (GE Healthcare).

For LC-MRM based detection of FC5, a sensitive and specific method recently described for detection of V_H_Hs in body fluids was utilized [28]. Briefly, the FASP-extracted EMVs described above were reanalyzed using the LTQ Orbitrap® in SRM mode and monitor FC5-specific signatures. This involved selecting the precursor m/z of 844.92 for FC5-specific peptide ITWGGDNTFYSNSVK and monitoring signature fragment ions 534.48, 729.47, 737.89, 1288.44. For quantification analysis, raw files generated by LTQ were converted to mzXML format and intensities were extracted using an in-house software Q-MRM, a modified version of MatchRx software [27].

## Results

### Proteomics of HBEC-EMVs

**Figure 2:**
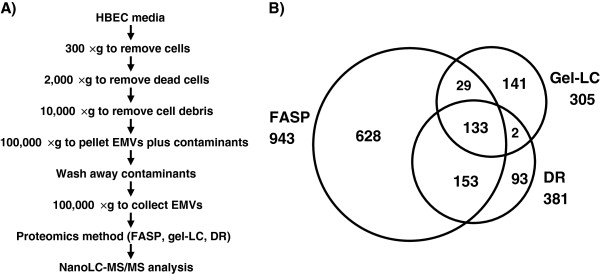
**Proteomics of human brain endothelial cell extracellular microvesicles (HBEC-EMVs).** (**A**) Workflow of the EMV isolation from HBEC media. (**B**) Venn diagram showing the overlap among the identified proteins by 3 proteomics methods used for analysis of EMVs. FASP: filtered-aided sample preparation, gel-LC: gel electrophoresis followed by nanoLC-MS/MS, DR: detergent removal. See Methods for more details.

EMVs were isolated from HBEC using the method shown schematically in Figure 2A; the protocol included extensive washing to minimize cell debris, artifacts and contaminating proteins. Three proteomics methods were then used for the molecular analysis of the isolated EMVs. These included gel-LC (1D-SDS-PAGE-LC-MS/MS) and gel-free methods, FASP and DR.

Figure 2B shows the number and overlap of proteins identified by each of these methods; 133 proteins were common amongst all three methods. The gel-free methods identified more proteins than the gel-LC and the overlap between the gel-free methods was also the highest (286 proteins); the FASP method identified the highest number of proteins. Since many proteins were still specific to gel-LC and DR methods, the gel-free and gel-based methods were considered complementary. In all, a total of 1179 proteins were identified in the EMVs of the immortalized HBEC using proteomics.

### Are HBEC-EMVs proteins identified by proteomics intact proteins?

**Figure 3:**
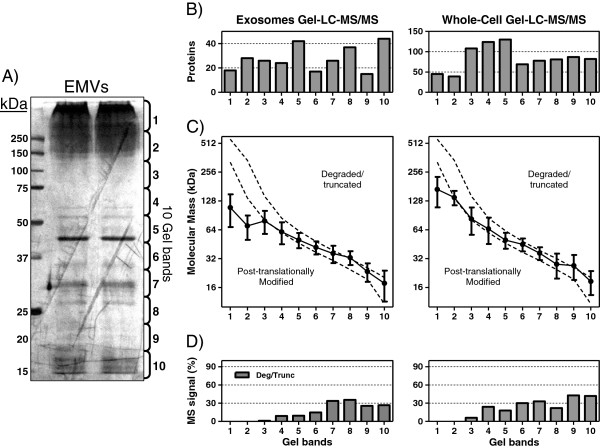
**Gel-LC MS/MS of HBEC-EMV proteins.** (**A**) Coommassie blue-stained gel following SDS-PAGE analysis of EMVs from HBEC. Shown are the molecular weight (MW) markers (left) and the position of ten bands that were cut out (right). (**B**) Total number of proteins identified in each band from SDS-PAGE of EMVs (left) or whole-cell HBEC extract (right). (**C**) Average theoretical MW (± SD) of the proteins in each gel band. The dotted lines represent the observed MW range of gel band, as determined by marker lane. If the theoretical MW is above the upper dotted line, it is likely degraded/truncated, whereas if it is below the lower dotted line, it is potentially post-translationally modified. Note the log scale. (**D**) Percentage of MS signal originating from degraded/truncated proteins in each gel band as described in panel C.

Since EMVs (especially exosomes) are known to originate from the cellular endocytic pathway that could include lysosomes and their digestive enzymes, there was a possibility that the EMVs may contain a large number of degraded proteins. To assess whether proteins identified from HBEC-EMVs by above methods are intact proteins, we examined if they separated on 1D-SDS-PAGE according to their expected molecular weight (MW). After separation of EMV proteins on 1D-SDS-PAGE, the entire lane was cut into ten sequential bands for in-gel digestion followed by nanoLC-MS/MS analysis to identify proteins in each gel band (Figure 3A). For comparison, HBEC-whole-cell extracts (WCEs) were also similarly analyzed by gel-LC. Shown in Figure 3 are the total number of proteins identified by gel-LC (Figure 3B) and the average expected (theoretical) MWs of the proteins in each gel band (Figure 3C) in HBEC-EMVs and HBEC-WCEs. Also shown are the observed MWs of the proteins (Figure 3C, dotted lines), as estimated from the MW markers (Figure 3A). The results show that the majority of proteins ran at or above their expected MW suggesting that they are not degraded or truncated. Only about 20% of the MS signal in HBEC-EMVs (Figure 3D) originated from degraded proteins. These results were closely comparable to gel-LC of WCEs (Figure 3C), which show about 24% of the MS signal (Figure 3D) from degraded proteins.

The proteins identified in the top two bands of gel-LC of HBEC-EMVs were found to run at significantly higher MW than expected and, for some, higher than their observed MW in WCEs. Most of these proteins are known glycoproteins (including adhesion molecules and other membrane proteins). This observation suggests that the proteins in bands 1 and 2 of HBEC-EMVs are potentially glycosylated, a post-translational modification that can stabilize proteins from degradation, especially against digestive enzymes in the lysosomes.

### EMV-specific and HBEC-EMV-specific markers

**Figure 4:**
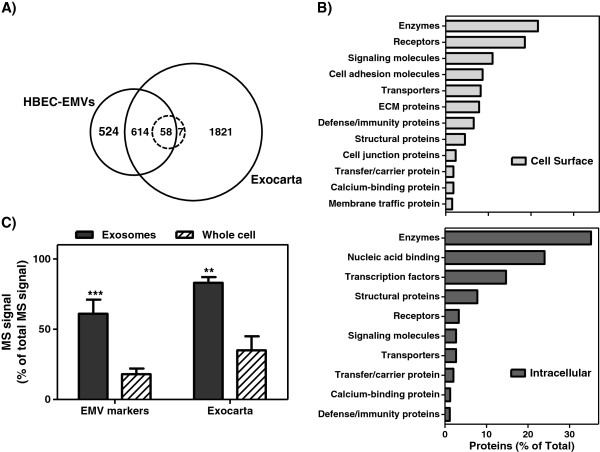
**EMV-specific and HBEC-EMV-specific markers (A) Venn diagram showing overlap among HBEC-EMVs, known 65 EMV markers (dotted circle), and ExoCarta proteins.** (**B**) Subclassification of the 524 HBEC-EMV-specific proteins (panel A). (**C**) Percentage of total MS signal originating from proteins in common with known 65 EMV markers or ExoCarta in HBEC-EMVs and whole HBEC extracts. The ** corresponds to *p* < 0.01 and *** corresponds to *p* < 0.001 for Mann Whitney *U*-test with n = 3.

More than 60 known markers of EMVs have been previously described [14]. In addition, >2000 proteins associated with *Homo sapiens* have been identified in ExoCarta, a large proteomics database of exosomes and EMVs in various human cell types [15]. To demonstrate that the HBEC-EMVs isolated by described methods are pure and contain known EMV-specific markers, we compared the 1179 identified HBEC-EMV proteins against the 60 known EMV markers in the ExoCarta database. As shown in Figure 4A, the majority of known exosome markers (58 of 65, Table 1) and many other ExoCarta-catalogued proteins were detectable in HBEC-EMVs. Importantly, 524 proteins identified in HBEC-EMVs did not overlap with proteins in ExoCarta, suggesting that they may be HBEC-specific. These signatures consisted of 35% cell-surface and 65% intracellular proteins and were further classified using Gene Ontology and Panther classification system (Figure 4B).

**Table 1:** Proteins identified in HBEC-EMVs by proteomics that were in common with known markers of exosomes*

**Protein category**	**Gene symbol**	**No. of Families detected**
(1) Antigen-presentation		
HLA class I histocompatibility antigen	HLA	2
(2) Cell adhesion		
Lactadherin	MFGE8	1
Thromospondin-1	THBS1	1
Integrins	ITG	5
(3) Cell structure and motility		
Actins	ACT	12
α-Actinin-4	ACTN	2
Cofilin-1	CFL1	1
Moesin	MSN	1
Myosin, heavy	MYH	5
Myosin, light	MYL	3
Radixin	RDX	1
Tublins	TUB	13
(4) Heat shock proteins and chaperones		
Heat shock cognate 71 kDa protein	HSPA8	1
T-complex protein 1	CCT	8
(5) Metabolic enzymes		
Aldolase A	ALDOA	1
Fatty acid synthase	FASN	1
Glyceraldehyde-3-phosphate dehydrogenase	GAPDH	1
Phosphoglycerate kinase 1	PGK1	1
Phosphoglycerate mutase 1	PGAM1	1
Pyruvate kinase isozymes M1/M2	PKM2	1
(6) Multi vesicular body (MVB) biogenesis		
Alix	PDCD6IP	1
ESCRT I complex		
Tumor susceptibility gene 101 protein	TSG101	1
Vacuolar sorting protein 28	VPS28	1
Vacuolar protein sorting-associatedprotein 37	VPS37	3
ESCRT II complex		
Vacuolar protein-sorting-associated protein 25	VPS25	1
Vacuolar protein-sorting-associated protein 36	VPS36	1
Vacuolar-sorting protein SNF8	SNF8	1
ESCRT III complex		
Charged MVB proteins	CHMP	2
(7) Signaling proteins		
14-3-3 Proteins	YWHA	6
GTPase HRas	HRAS	1
Rho GDP-dissociation inhibitor 1	ARHGDIA	1
Rho-related GTP-binding protein RhoC precursor	RHOC	1
Ras-related protein Rap-1b	RAP1B	1
Ras-related protein R-Ras2	RRAS2	1
Ras GTPase-activating-like protein	IQGAP1	1
Syntenin-1	SDCBP	1
Transforming protein RhoA	RHOA	1
Guanine nucleotide-binding protein (G proteins)		
-G(I)/G(S)/G(T) subunit beta	GNB	4
Protein category and description Gene symbol		
-G(I)/G(S)/G(O) subunit gamma	GNG	1
-G(S) subunit alpha	GNAS	1
-subunit alpha	GNA	4
-G(I), alpha	GNAI	1
(8) Tetraspanins		
CD9 antigen	CD9	1
CD63 antigen	CD63	1
CD81 antigen	CD81	1
CD82 antigen	CD82	1
(9) Transcription and protein synthesis		
Histones	HIST	14
Ribosomal proteins	RPS	34
Ubiquitin	RPS27A	1
Elongation factor 1-a 1	EEF1A1	1
(10) Trafficking and membrane fusion		
Annexins	ANXA	1
ADP-ribosylation factor	ARF	3
AP-2 complex subunit α-1	AP2A1	1
AP-2 complex subunit β-1	AP2B1	1
Clathrin heavy chain 1	CLTC	1
Rab GDP dissociation inhibitor b	GDI2	1
Ras-related protein Rab	RAB	9
Synaptosomal-associated protein 23	SNAP23	1

* The known markers of exosomes were obtained from Simpson *et al*[14]. For many proteins, more than one family member was detected.

We also examined the percentage of the MS signal originating from the known exosome proteins identified in HBEC-EMVs (Table 1). As shown in Figure 4C, >55% of the MS intensities was attributable to known 60 EMV markers and their families in HBEC-EMVs, a 3.3-fold higher than the signal from same proteins in HBEC-WCEs (*p* < 0.001, Mann Whitney *U*-test). Similarly, about 80% of the signal in HBEC-EMVs originated from ExoCarta-catalogued proteins, which was also significantly higher than the signal in HBEC-WCEs (2.4-folds, *p* < 0.01, Mann Whitney *U*-test). These results suggest that HBEC-EMVs obtained by described methods are highly enriched with known markers of exosomes, but also harbour many protein signatures that are specific to HBEC.

### HBEC-EMVs as vehicles of cell-cell communication

**Figure 5:**
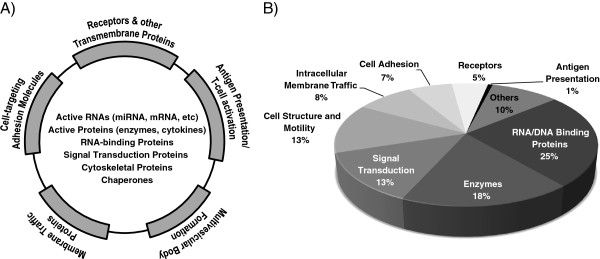
**Subclassification of surface and intravesicular molecules in EMVs.** (**A**) A ‘generic’ molecular map of EMVs. (**B**) Protein classification of HBEC-EMVs using Gene Ontology and Panther.

Communication between cells, including horizontal transfer of RNAs and proteins, is the main physiological role of EMVs; the ‘generic’ map of surface molecules and intravesicular content of EMVs (Figure 5A) uniquely reflects their function in cell-cell communication.

To examine whether the identified proteins in HBEC-EMVs have known roles associated with cell-cell communication, the 1179 identified proteins were categorized based on their known functional and biological classes using a combination of Gene Ontology database and Panther classification system. Most of the proteins could be classified into 8 key categories as shown in Figure 5B. These included key biological processes such as intracellular trafficking, signal transduction, cell adhesion, and cell motility. In addition, they included functional classes such as RNA/DNA-binding proteins, receptors, structural proteins and enzymes. A number of these categories were determined to be statistically over-represented when the 1179 proteins were compared to one-hundred random lists of 1179 proteins sampled from Uniprot human database. The classes overrepresented included membrane traffic proteins (p < 0.001), RNA/DNA-binding proteins (p < 0.001), cytoskeletal structural proteins (p < 0.001), and enzymes (p < 0.01).

To assess whether BEC-EMVs are capable of interacting with cells of the CNS, we analysed *in silico* whether the surface molecules on HBEC-EMVs could form protein-protein interactions with cell-surface molecules on astrocytes and neurons using *in situ* cell-cell interactomics approach, recently described by us for HBEC and Th17 cells [29]. The data was obtained from (in house) proteomics maps of primary human astrocytes (unpublished data) and published proteome of mouse cortical neurons [30]. The analysis found that 21 of the surface proteins of HBEC-EMVs could interact with 30 cell-surface proteins of human astrocytes forming 58 theoretical protein-protein interactions. Similarly, 35 HBEC-EMV surface proteins could interact with 39 neuronal surface proteins forming 87 theoretical protein-protein interactions. While these identified interactions are hypothetical and will require validation in co-culture assays, they indicate that EMVs released from HBECs can potentially interact as cell communication vesicles with both primary astrocytes and cortical neurons.

### Receptor-mediated transcytosis receptors in HBEC-EMVs

**Figure 6:**
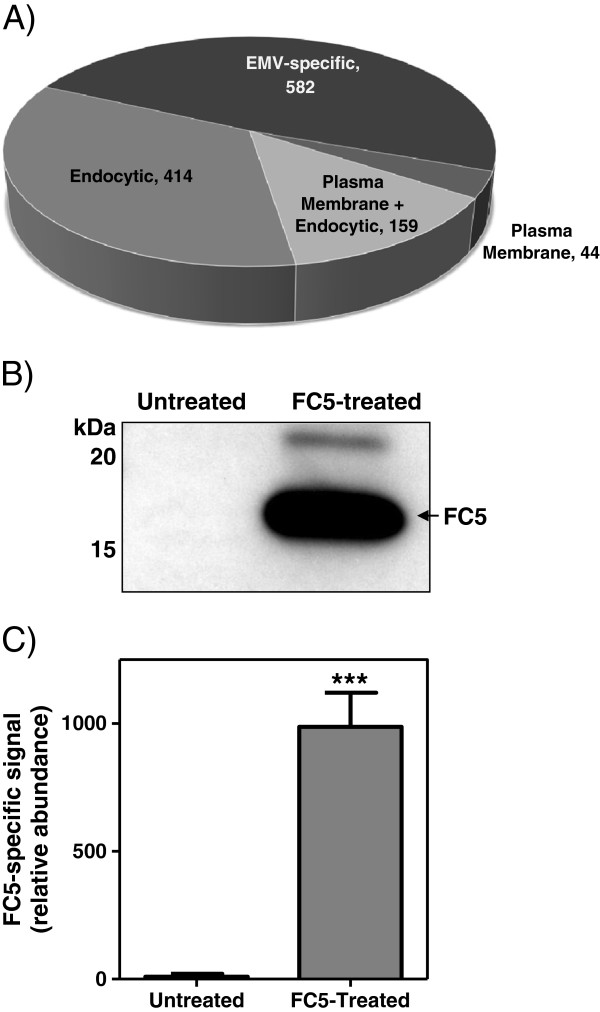
**HBEC-EMV origin and receptor-mediated transcytosis (RMT) receptors.** (**A**) Number of proteins in HBEC-EMVs that are in common with HBEC endocytic proteins and/or HBEC plasma membrane proteins. Most of the RMT receptors shown in Table 2 were in common with plasma + endocytic membranes. (**B**) Detection of FC5 in HBEC-EMVs from either control cells or cells treated with 5 μg/mL of FC5 for 24 h in serum-free conditions using Western blot analysis with polyclonal anti-V_H_H antibody (obtained from Biogen Idec, Cambridge, MA, USA). Equal volume amounts were loaded and the blot is a representative blot of n > 3. (**C**) Levels of FC5-specific peptide detected by MRM as described in Methods. The levels are relative to median “untreated” values. *** represents *p* < 0.001 for Mann–Whitney *U*-test with n = 3.

Several BBB-expressed receptors are known to undergo a receptor-mediated transcytosis (RMT). To examine whether these receptors could be found in HBEC-EMVs, we first compared 1179 proteins identified in HBEC-EMVs with proteins identified in plasma membranes and endocytic membranes of HBEC. As shown in Figure 6A, about 50% of the HBEC-EMV proteins were common with those identified in either endocytic- or plasma membranes of HBEC. Endocytic pathway proteins were shown to contribute to >30% of all proteins identified in HBEC-EMVs. We next examined whether known RMT receptors– especially those previously explored for therapeutic drug delivery across the BBB – were also present in the HBEC-EMVs. As shown in Table 2, several known receptors for BBB ‘Trojan horses’ were found in HBEC-EMVs, including TMEM30A, a putative antigen for the single-domain antibody, FC5, shown to transmigrate the BBB *in vitro* and *in vivo*[31-33](manuscript submitted). To confirm that FC5 could be ‘shuttled’ by HBEC-EMVs, the RMT was initiated by the addition of FC5 to HBEC, and EMVs collected from these cells were analysed by proteomics and Western blotting. FC5 was clearly detectable in the EMVs by Western blotting using polyclonal anti-V_H_H antibody (Figure 6B). The presence of FC5-specific signal in BEC EMVs was additionally confirmed and quantified (Figure 6C) using SRM-ILIS method as recently described [28]. The presence of known RMT receptors and co-localization of FC5 with its putative RMT receptor, TMEM30A, in HBEC-EMVs suggest that these vesicles might be implicated in RMT process across the BBB.

**Table 2:** Known BBB RMT receptors identified in HBEC-EMVs

**Receptor**	**BBB-Crossing Ligand**	**References**
Transferrin Receptor (TFRC)	OX26 mAb	[2]
Insulin Receptor (INSR)	83-14 mAb	[37]
Low-Density Lipoprotein Receptor (LDLR)	C7 mAb	[38]
LDLR-related proteins (LRP1, LRP1B, LRP2)	P97 proteins and Angiopep peptides	[6,39]
Cell cycle control protein 50A (TMEM30A/CDC50A)	FC5 sdAb	[31-33]
Fc-Binding Proteins (e.g., FcγBP)	Fc-containing antibodies	

## Discussion

This manuscript details methods for isolation as well as sensitive MS-based protocols for molecular analyses of EMVs from HBEC. Using these methods, 1179 unique proteins were identified in HBEC-EMVs. These methods, in combination with bioinformatics tools, were used to demonstrate that the isolated HBEC-EMVs (i) are not artifacts and contain intact, potentially post-translationally modified proteins, (ii) contain a majority of known exosome-specific proteins, as well as unique ‘signature’ proteins, (iii) contain proteins implicated in receptor-mediated transcytosis across the BBB.

### Are EMVs artifacts?

EMVs were originally believed to be cellular artifacts and thought of as mechanisms through which cells discard inert debris [10,30]. Many reports have since shown that EMVs are real, released cellular sub-compartments that consist of subsets of few protein families.

The BEC EMVs isolated by the described differential centrifugation method have not been morphologically characterized and may include both small (100 nm) and larger (up to 1000 nm) EMVs. Proteomic analyses of these EMVs reported in this study confirmed that HBEC-EMVs contain specific sub-sets of intact proteins, originating from the plasma membrane, endocytic pathway(s) and the cytosol. A subgroup of higher molecular weight proteins represented in EMVs appear to be post-translationally modified, compared to same proteins in whole-cell extracts, suggesting that they may originate from compartments characterized by high glycosylation, such as BEC luminal membranes or endocytic vesicles(s).

### Specificity of HBEC-EMVs

The diagnostic potential of EMVs has been aggressively investigated [12,13], since they contain tissue and disease-specific biomarker signatures [9,21]. The tissue-specificity of EMVs is determined by specific RNA sequences and specific cell-surface molecules. The BBB-specific EMVs in body fluids could contain biomarkers useful for diagnosis or monitoring of brain diseases, since they could be ‘shed’ into the circulation from luminal membranes of BEC and potentially shuttled across the BBB from the abluminal side. We have found that about 20% of the HBEC-EMVs MS signal originated from proteins that were absent in exosomes from other cell types, suggesting that these proteins are potentially unique to HBEC-EMVs. Some of these included cell-surface proteins, including adhesion molecules and other cell-cell interacting molecules (Figure 4B).

The molecular signatures of EMVs can change under different biological conditions (*in vitro* insults or diseases state) [9,21]. For example, we have observed that HBEC-EMVs molecular profile changed significantly in response to inflammatory insults (unpublished data). Therefore, monitoring HBEC-EMV-specific and disease-modified RNAs, proteins, glycoproteins and glycans in blood-derived EMVs by targeted ‘omics’ has potential diagnostic significance for CNS disorders. However, the utility of BBB EMVs as a source of disease-specific biomarkers remains to be validated in further *in vitro* and *in vivo* studies.

### HBEC-EMVs as a vehicle for cell-cell communications in CNS

The cell-cell communication mediated by EMVs occurs predominantly by two processes: surface contact of vesicles with cells triggering donor cell signaling pathways, and/or delivery of vesicle content into the recipient cell (endogenous transduction). Consistent with these roles in cell-cell communication, the surface of EMVs is typically enriched in cell-targeting/adhesion molecules (e.g., tetraspanins and integrins), membrane trafficking proteins, proteins involved in MVB formation, antigen-presenting molecules (e.g., MHC class I and class II), and membrane cytokines, whereas their luminal content mainly consists of functionally-active RNAs (e.g., mRNA, microRNA, viral RNA), RNA-binding proteins, ribosomes, functionally-active proteins including enzymes (e.g., metalloproteases, metabolic enzymes) and cytokines (Figure 5A). HBEC-EMVs molecular make-up is consistent with this ‘generic’ exosome composition.

Given tight anatomical and functional integration of the cellular elements of the neurovascular unit, including BEC, pericytes, astrocytes and neurons, we surmise that BEC exosomes could play similar roles in transducing information among the cells in the neurovascular unit. The emerging role of neuronal exosomes in neuronal-glial communication and inter-cellular transfer of signaling miRNAs contributing to neuronal development and disease mechanisms has recently been reviewed [22]. The *in silico* interactomics analyses confirmed that, based on molecular profile of HBEC-EMVs, they could engage in numerous cell-surface interactions with both astrocytes and neurons. Similar EMV-mediated communication could occur among BEC and peripheral inflammatory cells during processes of immune surveillance, rolling, adhesion and transmigration.

### Are HBEC-EMVs BBB ‘transcytosing’ vesicles?

The first discovery of exosomes, almost three decades ago, involved detection of anti-TFRC antibody by electron microscopy in reticulocytes (summarized by Thery *et al*[34]) in the following order: (i) on the surface of the cells and clathrin-coated pits, (ii) inside early endosomes, (iii) on the surface of internal vesicles of multivesicular endosomes, and finally (iv) on the released exosomes after fusion of the multivesicular endosomes with the plasma membrane. The RMT pathway and exosome formation have notable similarities. The HBEC-EMVs contained several receptors previously shown to carry macromolecules across the BBB via RMT, including TFRC, LRPs, LDLR, INSR and TMEM30A (Table 2). A hypothetical pathway by which these receptors and their ligands are ‘sorted’ into HBEC exosomes during luminal-abluminal RMT process is shown in Figure 1. A similar process may theoretically occur in the opposite direction, resulting in RMT receptor recycling, or ‘transfer’ of parenchymal exosomes into the circulation. The presence of known BBB RMT receptors in HBEC-EMVs might suggest that, among 524 ‘unique’ proteins identified in HBEC-EMVs, there may be additional novel and more specific RMT receptors exploitable for delivery of macromolecules across the BBB.

Interestingly, after the addition of RMT-triggering antibody FC5, we observed both a 4-fold increased amount of EMVs being produced by HBEC (based on total LC-MS signal; not shown) and presence of FC5 in these EMVs. This suggests that, under specific conditions, brain endothelial cells could regulate the amount of EMVs produced and ‘shed’ into abluminal or circulatory space.

### EMVs as BBB drug-delivery vehicles

The possibility of using exosomes as drug-delivery vehicles, in particular for gene therapy with siRNAs, has gained significant attention in recent literature. In the study by Alvarez-Erviti *et al*[35], autologous exosomes derived from dendritic cells engineered to express exosomal membrane protein Lamp2b fused to the neuron-specific RVG peptide, were loaded with exogenous siRNA and shown to transduce brain parenchymal cells knocking-down the therapeutic target, BACE1, after systemic injection. Exosomes were also attempted as intranasal delivery vehicle for anti-inflammatory drugs [36]. The advantage of self-derived exosomes over other lipid-based nanocarriers is that they are immunologically inert and are thought to possess ‘intrisic ability’ to cross biological barriers. Although this assertion requires further confirmation, the possibility remains that tissue-specificity of delivery could be improved by using homologous tissue exosomes. Therefore, HBEC-EMVs could potentially be exploited as brain-selective nanocarriers for therapeutic delivery across the BBB.

## Conclusions

The first comprehensive evaluation and cataloguing of proteins expressed in EMVs derived from brain endothelial cells described in this manuscript, demonstrated that these vesicles contain common proteins typical of exosomes from different tissues, as well as proteins that may be specific to unique functions of brain endothelial cells within the context of the neurovascular unit, including the transport of solutes and biologics across the blood–brain barrier.

## Abbreviations

BEC: Brain endothelial cell; CNS: Central nervous system; DR: Etergent removal; EMV: Extracellular microvesicles; FASP: Filtered-aided sample preparation; gel-LC: Gel electrophoresis followed by nanoLC-MS/MS; HBEC: Human BEC; LC: Liquid chromatrography; MS: Mass spectrometry; MS/MS: Tandem MS; MVB: Multivesicular bodies; NanoLC: Nanoliter flow LC; NVU: Neurovascular unit; RMT: Receptor-mediated transcytosis; TFRC: Transferrin receptor; WCE: Whole-cell extract.

## Competing interests

The authors declare that they have no competing interests.

## Authors’ contributions

**ASH** Designed studies, developed proteomic methods, interpreted data, conceptualized and wrote the manuscript. **CED** Cultured and maintained cells, isolated EMVs, carried out western blotting and proteomics on EMVs. **TLT** Analyzed EMVs using FASP method. **CS** Initial help with analysis of EMV isolation, characterization and validation. **JKS** Provided expertise/guidance with isolation of EMVs. **DBS** Provided general guidance, contributed to writing and revising the manuscript. All authors have read and approved the final version of the manuscript.
